# Graph Laplacian-based spectral multi-fidelity modeling

**DOI:** 10.1038/s41598-023-43719-1

**Published:** 2023-10-03

**Authors:** Orazio Pinti, Assad A. Oberai

**Affiliations:** Aerospace and Mechanical Engineering Department, University of Southern California, Los Angeles, 90007 USA

**Keywords:** Computational science, Mechanical engineering

## Abstract

Low-fidelity data is typically inexpensive to generate but inaccurate, whereas high-fidelity data is accurate but expensive. To address this, multi-fidelity methods use a small set of high-fidelity data to enhance the accuracy of a large set of low-fidelity data. In the approach described in this paper, this is accomplished by constructing a graph Laplacian from the low-fidelity data and computing its low-lying spectrum. This is used to cluster the data and identify points closest to the cluster centroids, where high-fidelity data is acquired. Thereafter, a transformation that maps every low-fidelity data point to a multi-fidelity counterpart is determined by minimizing the discrepancy between the multi- and high-fidelity data while preserving the underlying structure of the low-fidelity data distribution. The method is tested with problems in solid and fluid mechanics. By utilizing only a small fraction of high-fidelity data, the accuracy of a large set of low-fidelity data is significantly improved.

## Introduction

Multi-fidelity methods have been widely used in different areas of science and engineering, including optimization^1,2^, uncertainty quantification^3^, uncertainty propagation^4,5^, and statistical inference^6^ (see Fernández-Godino et al.^7^ and Peherstofer et al.^5^ for two comprehensive reviews). The fundamental idea behind these methods is to combine a large amount of low-fidelity data, which is relatively inexpensive to compute or measure, with a much smaller set of high-fidelity data acquired at higher cost. The objective of multi-fidelity modeling is to improve the accuracy of the large low-fidelity data set by utilizing the small set of high-fidelity data.

In a typical multi-fidelity framework, low-fidelity data is acquired to obtain an approximation of the response of the system. Then, a limited number of high-fidelity data points are computed or measured. Finally, techniques that learn the response from the low-fidelity data, and improve it by using the high-fidelity data are applied. Co-kriging methods have been extensively investigated in this context^8–11^, where the multi-fidelity response is expressed as a weighted sum of two Gaussian processes, one modeling the low-fidelity data, and the other representing the discrepancy between the low- and high-fidelity data. The parameters of the mean and correlation functions of these processes are determined by maximizing the log-likelihood of the available data.

Other methods make use of radial basis functions (RBFs) to model the low-fidelity response. Specifically, the low-fidelity surrogate is written as an expansion in terms of a set of RBFs, and the coefficients are determined by interpolating the available low-fidelity data. The multi-fidelity approximation is then obtained in different ways. These include determining a scaling factor and a discrepancy function, which can be modeled using a kriging surrogate^11^, or another expansion in terms of RBFs^12,13^. In some cases the multi-fidelity surrogate is constructed by mapping the low-fidelity response directly to the high-fidelity response^14^.

More recently, deep neural networks have been used to fit low-fidelity data and learn the complex map between the input and output vectors in the low-fidelity model. Then, the relatively small amount of high-fidelity data is used in combination with techniques such as transfer learning^15,16^, embedding the knowledge of a physical law through physics-informed loss functions^17–20^, or, in the case of multiple levels of fidelity, concatenating multiple neural networks together^21^. An approach that involves training a physics-constrained generative model, conditioned on the low-fidelity snapshots, to produce solutions that are higher-fidelity and higher-resolution has also been proposed^22^.

Another class of methods, suitable when the response of the system consists of a high-dimensional vector, first performs order-reduction using the low-fidelity data, and then inject accuracy using the high-fidelity data in a reduced-dimensional latent space. This has been accomplished by computing the low- and high-fidelity proper orthogonal decomposition (POD) manifolds, aligning them with each other, and replacing the low-fidelity POD modes with their high-fidelity counterparts^23^. This has also been done by first solving a subset selection problem to construct a surrogate model of the low-fidelity response in terms of a few important snapshots, then generating their high-fidelity counterparts, and finally using these in the multi-fidelity surrogate model^24–26^.

In contrast to the methods described above, the approach developed in this manuscript relies on the spectral properties of the graph Laplacian constructed from the low-fidelity data. It uses these properties to determine the points at which high-fidelity data ought to be acquired, and to embed the structure of the low-fidelity data into the multi-fidelity model. It also differs from most co-kriging and RBFs-based methods in how it treats outputs with multiple quantities of interest. While most methods tend to ignore the joint distribution of these quantities, the proposed method explicitly utilizes it in constructing the multi-fidelity response. The proposed approach also has strong connections with semi-supervised classification algorithms on graphs^27–31^, and relies on theoretical results on consistency of graph-based methods in the limit of infinite data^32,33^.

**Figure 1:**
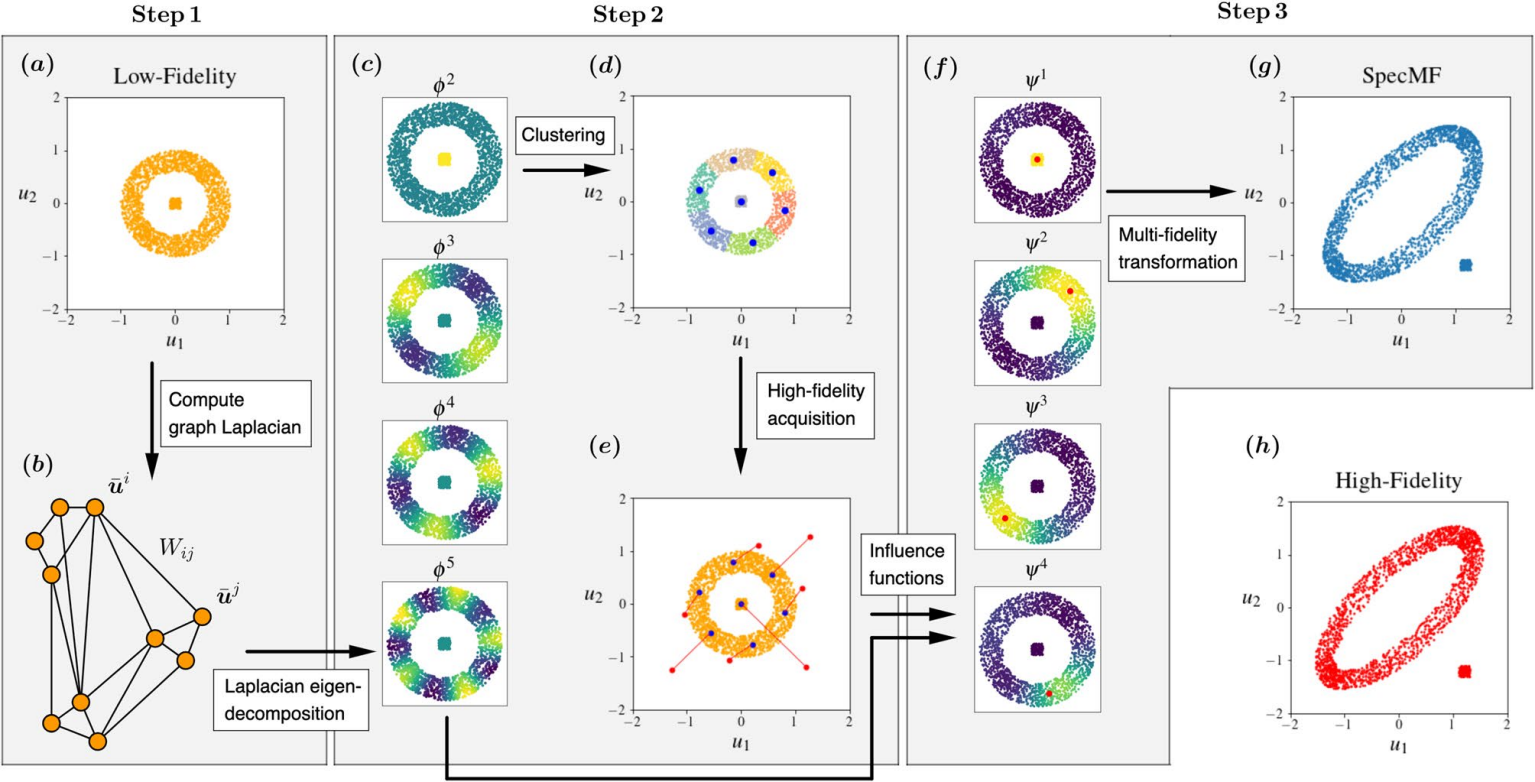
Workflow for the spectral multi-fidelity (SpecMF) method applied to an illustrative problem. (**a**) Generate low-fidelity data. (**b**) Compute a graph Laplacian using the low-fidelity data. (**c**) Compute the eigen-decomposition of the graph Laplacian. (**d**) Perform spectral clustering of the low-fidelity data and find the points closest to the clusters centroids. (**e**) Acquire high-fidelity data only for these points. (**f**) Solve a convex minimization problem to find one influence function for each point with a high-fidelity counterpart. The influence functions are constructed from the low-lying eigenfunctions of graph Laplacian. (**g**) Generate the multi-fidelity approximation of the data set. (**h**) For this illustrative example, this is compared with the corresponding high-fidelity data set.

### Overview of the methodology

Given a parametric physical problem, we denote by $\varvec{\mu }\in \mathbb {R}^P$ the vector of input parameters, and by $\bar{\varvec{q}} (\varvec{\mu }) \in \mathbb {R}^Q$ and $\varvec{q}(\varvec{\mu }) \in \mathbb {R}^Q$ the low- and high-fidelity output vectors, respectively. These vectors represent a set of *Q* output quantities of interest of the problem. Low- and high-fidelity data points, denoted by $\bar{\varvec{u}}\in \mathbb {R}^D$ and $\varvec{u}\in \mathbb {R}^D$, respectively, with $Q \le D \le Q+P$, are constructed from components of input parameters and output quantities. That is, $\bar{\varvec{u}}= \varvec{R} (\varvec{\mu }, \, \bar{\varvec{q}})$ and $\varvec{u}= \varvec{R} (\varvec{\mu }, \, \varvec{q})$, where $\varvec{R}$ is a restriction operator that extracts the appropriate components of the input parameters and output quantities of interest. The choice of $\varvec{R}$ is problem dependent and is described in Supplementary Appendix H. Two obvious choices are when the data comprises all input and output components, that is, $\varvec{R} (\varvec{\mu }, \, \varvec{q}) = [\varvec{\mu }, \, \varvec{q}]$, and when it comprises only of the output components, that is, $\varvec{R} (\varvec{\mu }, \, \varvec{q}) = \varvec{q}$.

The multi-fidelity method combines a dense set of low-fidelity data points with a few, select, high-fidelity points to generate a dense set of multi-fidelity points. The steps to accomplish this are outlined in Fig. 1 in the context of a two-dimensional illustrative example. Specifically, a large set of low-fidelity data $\{ \bar{\varvec{u}}^{i}\}_{i=1}^{\bar{N}}$ is generated for different instances of input parameters $\{ \varvec{\mu }^i\}_{i=1}^{\bar{N}}$. These data points are treated as the nodes of a weighted graph with weight matrix $\varvec{W} \in \mathbb {R}^{\bar{N}\times \bar{N}}$ in the normalized data coordinates, and a normalized graph Laplacian $\varvec{L} = \varvec{D}^{-1/2} (\varvec{D} - \varvec{W}) \varvec{D}^{-1/2}$ is evaluated. Here $\varvec{D}$ is a diagonal matrix whose entries are the row-sum of the weight matrix $\varvec{W}$. The low-lying eigen-spectrum $(\lambda _i,\,\varvec{\phi }^i),\, i=1,\,\dots ,\,K$, of the graph Laplacian is computed and used to cluster the low-fidelity data by employing K-means clustering in the eigenfunction coordinates. Data points that are closest to the centroids of the clusters are determined (marked as blue dots in Fig. 1) and their high-fidelity counterparts $\{\varvec{u}^{i}\}_{i=1}^{\bar{N}}$, $N\ll \bar{N}$, are evaluated. The low-lying eigenfunctions of the graph Laplacian are used once again to construct a set of influence functions $\varvec{\psi }^{(i)}$, $i=1,\,\dots ,\,N$, which are expressed as linear combination of the eigenfunctions followed by a soft-max transform. The mapping from low- to multi-fidelity data points is written as a linear combination of the influence functions times the displacement vector that maps each low-fidelity point to its high-fidelity counterpart. The coefficients linking the influence functions to the eigenfunctions are determined by solving a convex minimization problem which minimizes the distance between the transformed low-fidelity points and their high-fidelity counterparts while penalizing the use of eigenfunctions with large eigenvalues. This penalty term preserves the structure found in the low-fidelity data. This overall approach is referred to as Spectral Multi-Fidelity (SpecMF) method, since the spectral properties of the graph Laplacian are utilized to embed the structure of the low-fidelity data into the multi-fidelity model.

The proposed method is endowed with some desirable theoretical properties. These are described below and the results are derived in Supplementary Appendices A, B, and C.

#### Property 1

An explicit expression for the gradient of the loss function with respect to the optimization parameters can be computed, lowering the computational cost of the algorithm.

#### Property 2

In the limit of a small data misfit term (which happens as the optimization iterations converge), the Hessian of the loss function is positive definite. This proof is based on recognizing that (*i*) the data misfit term is in the form of a least-squares residual and (*ii*) the regularization term is a positive-definite quadratic form. This ensures that the resulting optimization problem is solved easily.

#### Property 3

Under the assumptions (*a*) the low-fidelity data is partitioned into *M* clusters, (*b*) the high-fidelity data differs from the low-fidelity data by distinct rigid translations applied to each cluster, and (*c*) the high-fidelity version of one point per cluster is known, the proposed approach permits a transformation that maps each low-fidelity point to the true high-fidelity point in the limit of infinite low-fidelity data and as the regularization parameter tends to zero. That is, the multi-fidelity data set converges to its high-fidelity counterpart. This is a consistency result that demonstrates the proposed method can solve this special problem exactly.

## Results

In what follows, we apply the proposed method to two numerical problems. The first is an application to linear elasticity, where the dimension of the input parameters space and the data space are $P=5$ and $D=5$, respectively. The second is a fluid dynamics problem, with $P=5$ and $D = 3$. Furthermore, for the elasticity problem, we also apply the SpecMF method to an entire field discretized on a grid with 100 points, so that $D = 100$. These problems were solved on a Apple M1 Pro processor with 8 cores, and the computational time for a fixed value of regularization parameter is around 1–2 min.

We compare our results with a co-kriging method^8,9^ applied to each output quantity of interest individually, i.e. $q_k=q_k(\varvec{\mu }),\, k=1,\,\dots ,\,Q$. In co-kriging, the multi-fidelity approximation $Z_k(\varvec{\mu })$ is constructed as a weighted sum of two Gaussian processes, $Z_k(\varvec{\mu }) = \gamma \bar{Z}_k(\varvec{\mu }) + Z_k^d(\varvec{\mu })$, where $\bar{Z}_k$ models the low-fidelity data $\{(\varvec{\mu }^i,\,\bar{q}_k^i)\}_{i=1}^{\bar{N}}$, $\gamma$ is a scaling factor, and $Z_k^d$ models the discrepancy between the high-fidelity data $\{(\varvec{\mu }^i,\,q_k^i)\}_{i=1}^{N}$ and $\gamma \bar{Z}_k$. For both these Gaussian processes the covariance function is an anisotropic Gaussian kernel, where different length scales are used for each coordinate of the input parameter space:

1 $$\begin{aligned} \textrm{cov}(\bar{Z}_k(\varvec{\mu }^i),\, \bar{Z}_k(\varvec{\mu }^j))&= \bar{\sigma }_k^2 \exp \bigg ( - \sum _{p=1}^P \frac{(\mu ^i_p - \mu ^j_p)^2}{\bar{l}_{k,\,p}^2}\bigg ), \end{aligned}$$

2 $$\begin{aligned} \textrm{cov}(Z^d_k(\varvec{\mu }^i),\, Z^d_k(\varvec{\mu }^j))&= \sigma _k^2 \exp \bigg ( - \sum _{p=1}^P \frac{(\mu ^i_p - \mu ^j_p)^2}{l_{k,\,p}^2}\bigg ). \end{aligned}$$

The hyper-parameters of these kernels, such as $\bar{\sigma }_k,\,\sigma _k,\,\bar{l}_{k,\,p},\,l_{k,\,p}$, the scaling factor $\gamma$ and the mean values of $\bar{Z}_k$ and $Z^d_k$, are computed by maximizing the log-likelihood of the data^9^. In our experience, this optimization problem could be very sensitive to the initial guess and the prescribed bounds in the search space. We addressed this issue by utilizing a grid search for the initial guesses to arrive at the best results. All computations were performed using the open-source computing platform OpenMDAO^34^.

### Traction on a soft material with a stiff inclusion

#### Problem description

**Figure 2:**
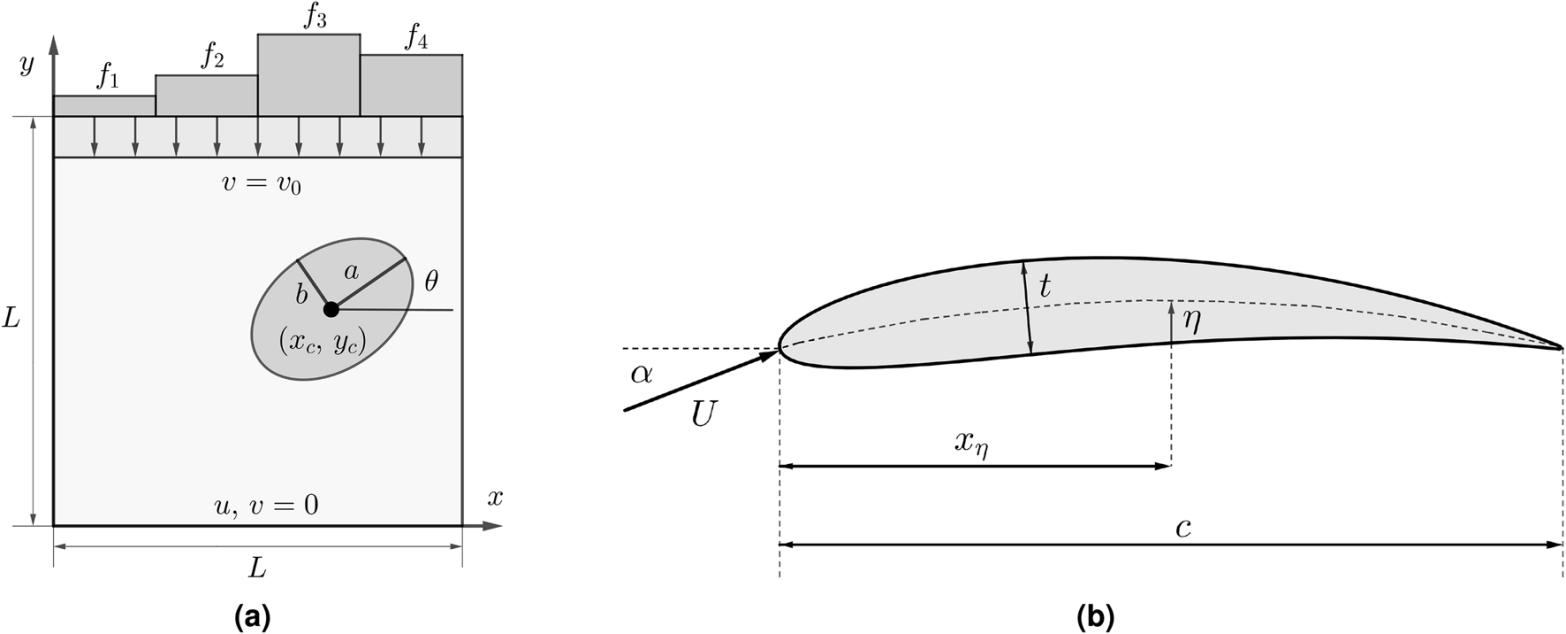
(**a**) Schematic of the soft body (light grey) with the elliptic stiffer inclusion (dark grey). The square is compressed on top with a uniform displacement $v=v_0$, while the bottom is fixed. The vertical traction is integrated over the top side across equal sections to compute the localized forces $f_i$. (**b**) Schematic of the airfoil with the input parameters.

We examine a problem of linear elasticity which involves a soft square sheet in plane stress with an internal stiffer elliptic inclusion. The length of the edge of the square is $L =10 \, \textrm{cm}$, its Young’s modulus is $E = 1 kPa$, and the Young’s modulus of the inclusion is $E_i = 4E$. Both the body and the inclusion are incompressible. The bottom edge of the square is fixed, and a uniform downward displacement $v_0 = -5 \, \textrm{mm}$ is applied to the top edge. The top edge is traction free in the horizontal direction while the vertical edges are traction-free in both directions (Fig. 2a). We wish to predict attributes of the vertical traction field on the upper edge as a function of the inclusion shape, orientation and location. This problem is motivated by the need to identify stiff tumors within a soft background tissue, which is particularly relevant to detecting and diagnosing breast cancer tumors^35,36^.

#### Parameters and quantities of interest

The input parameters of the problem are the coordinates of the center of the elliptical inclusion $(x_c,\,y_c)$, its orientation $\theta$, and its major and minor semi-axes *a* and *b* (see Fig. 2a). The minimum and maximum values for these parameters are reported in Table 1. The output quantities of interest include the values of the localized vertical forces on the top edge. These are determined by dividing the top edge into $M = 4$ sections of equal length and integrating the vertical traction $\sigma _{yy}$ over each section. This results in *M* values of localized forces $f_i, \; i = 1, \dots , M$ (see Fig. 2a),

3 $$\begin{aligned} f_i = \int \limits _{(i-1)\frac{L}{M}}^{i\frac{L}{M}} \sigma _{yy} (x,\, L) \textrm{d}x,\;\;\;\;i=1,\,\dots ,\,M. \end{aligned}$$

In addition to these forces, the maximum value of traction on the top edge is included as a quantity of interest. Therefore, the $M+1$ quantities of interest are $q_i = f_i, \; i=1,\,\dots ,\,M$, and $q_{M+1} = \max _x |\sigma _{yy}(x,\,L)|$. As the location, orientation and size of the inclusion is varied, the traction field on the top surface changes, which in turn changes the *M* components of the localized forces, and the maximum value of traction.

We consider the case where the data space is constructed only from the output vector, that is $\varvec{u}(\varvec{\mu }) = \varvec{q}(\varvec{\mu })$. The case of including the input vector in the data space, that is $\varvec{u}(\varvec{\mu }) = [\varvec{\mu }, \varvec{q}(\varvec{\mu })]$, yields comparable results and is described in Supplementary Appendix H.

**Table 1:** Range spanned by input parameters for the traction and airfoil problems.

Parameter	Min	Max	Units
Soft body with inclusion			
$x_c$	2.5	7.5	cm
$y_c$	5	7.5	cm
$\theta$	0	180	degree
*a*	1	2	cm
*b*	1	2	cm

Parameter	Min	Max	Units
Airfoil			
$\eta$	1	6	%c
$x_\eta$	4	7	$0.1 \times c$
*t*	10	20	%c
$\alpha$	− 5	12	degree
*Re*	$10^3$	$10^7$	1

#### Low- and high-fidelity models

We employ two finite element-based models that differ in the number of elements of the mesh. The low-fidelity model uses a coarse mesh with around 400 elements, whereas the high-fidelity model has a fine mesh with around 25,000 elements. It is verified that the high-fidelity model produces a solution that is mesh-converged.

#### Numerical results

We generate $\bar{N} = 1120$ samples of the input parameters by treating each parameter as an independent random variable that is uniformly distributed within its range. For each instance, we generate the low- and high-fidelity solutions. We use $N=30$ high-fidelity data points to construct the multi-fidelity results, and the remainder for testing the performance of method. We observe that the low-fidelity traction field captures the correct trend, but tends to underestimate the true magnitude (see Supplementary Appendix F).

**Figure 3:**
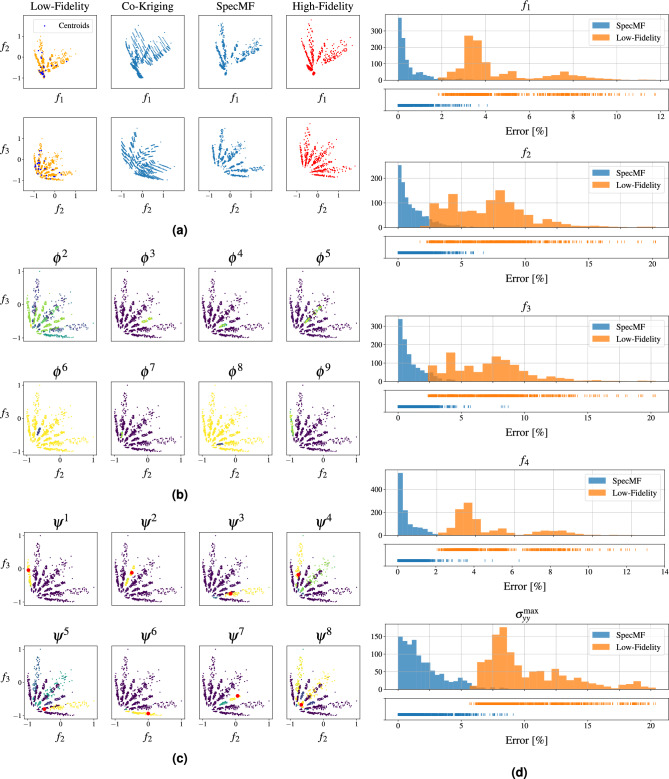
Results for the traction problem. (**a**) Low-fidelity, co-kriging, SpecMF and high-fidelity data. Low-fidelity data points are shown together with the points closest to the centroids of the clusters (in blue). (**b**) Eight eigenfunctions from the low-lying spectrum projected onto the $(f_2,\,f_3)$ plane. (**c**) Influence functions for eight control points projected onto the $(f_2,\,f_3)$ plane. (**d**) Error distribution for the low-fidelity and SpecMF model for each output component.

To visualize the five-dimensional data set, we project the data points in the $(f_1,\, f_2)$ and $(f_2,\, f_3)$ planes. In the first and fourth columns of Fig. 3a, we show a comparison between the scaled low- and high-fidelity data sets. We notice that low-fidelity data captures the structure of the high-fidelity data, however has a smaller spread.

The graph Laplacian is constructed from the low-fidelity data points; its low-lying eigenfunctions are shown in Fig. 3b in the $(f_2,\,f_3)$ plane. We observe that the eigenfunctions localize different regions of the low-fidelity data. Thereafter, we determine the coordinates of each low-fidelity data point in the eigenfunctions space and then perform K-means clustering to find the points closest to the centroids of $N=30$ clusters. These points are shown in blue in leftmost column of Fig. 3a, and correspond to the points where we utilize the high-fidelity data. We observe that these points appear to be evenly distributed over the span of the low-fidelity data. Next, we determine the influence functions for the multi-fidelity approximation by solving the minimization problem described in (17). The selection of the hyper-parameters is described in Supplementary Appendix F. A subset of the influence functions and the corresponding data points are shown in Fig. 3c. We observe that all the influence functions peak at their respective data point and vanish away from it.

The final multi-fidelity SpecMF approximation, which is generated via (14), is shown in third column of Fig. 3a. The results of the co-kriging method are also shown in the second column of the same figure. We observe that the SpecMF approximation appears to stretch the low-fidelity data distribution to make it closer to the high-fidelity distribution. In contrast, the co-kriging method appears to have distorted the underlying structure of the data.

To quantify the error in the SpecMF data $\varvec{w}^{i}$, we compute the relative absolute difference with respect to the high-fidelity data $\varvec{u}^{i}$ at each point *i* and for every component *k*,

4 $$\begin{aligned} e_k^i = \frac{| w^i_k - u^i_k |}{ E(|u_k|)} \times 100\%, \qquad i=1,\,\dots ,\,N_{val}, \quad k=1,\,\dots ,\,D. \end{aligned}$$

Here $E(\cdot )$ denotes the average over all validation points *i*, and $N_{val}$ is the number of validation points (in this case, $N_{val} = \bar{N}- N$). Similar errors are computed for the low-fidelity data and the co-kriging based multi-fidelity approximation. The histograms of the errors for the SpecMF and low-fidelity data are shown Fig. 3d for each output component. In each case we observe that the error distribution for the SpecMF data is more closely centered around zero, and presents a much smaller spread with respect to the distribution of the low-fidelity errors. The mean value of these errors is reported in Table 2 for each data component. We observe that the error for the low-fidelity data ranges between 5 and 10%. Co-kriging method reduces this error, but the SpecMF method is 1.5–2 times more accurate, improving the accuracy of the low-fidelity data by factor of 5–9 times.

**Table 2:** Error for the low-fidelity, co-kriging, and SpecMF data for each output component.

Error [%]	Soft body with inclusion						Airfoil		
Quantity of interest	$f_{1}$	$f_{2}$	$f_{3}$	$f_{4}$	$\sigma _{yy}^{\textrm{max}}$	$\sigma _{yy}$	$C_{L}$	$C_{D}$	$C_{M}$
Low-fidelity	4.48	7.15	7.21	4.65	10.19	6.95	40.40	28.84	216.96
Co-kriging	1.04	1.95	1.91	0.90	2.94	–	30.21	34.33	57.44
SpecMF	**0.52**	**1.2**	**1.06**	**0.51**	**2.0**	**1.52**	**18.56**	**10.77**	**45.95**
Improvement factor (SpecMF)	8.60	5.95	6.8	9.16	5.09	4.58	2.18	2.68	4.73

For the SpecMF the improvement factor, defined as the ratio of the low- and multi-fidelity error, is also reported.

Smallest values of error for each output component are in bold.

#### Multi-fidelity model for the entire traction field

**Figure 4:**
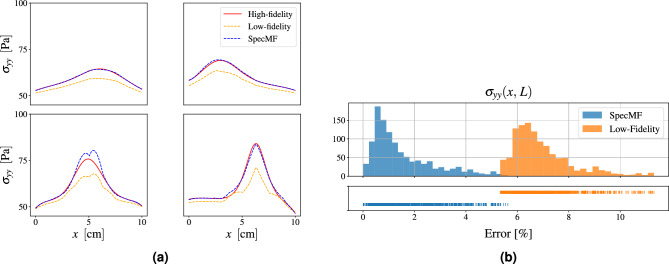
Results for the entire traction field problem. (**a**) Low-, high-, and multi-fidelity solutions for four test cases. (**b**) Error distribution for the low-fidelity and SpecMF solutions.

To test the method with a higher dimensional data space, we consider a modified version of the traction problem. Instead of computing the four localized forces and the maximum value of the traction on the top surface, that is $Q = D =5$, we use the entire traction field discretized over 100 points. Thus the dimension of the output and data vectors is $Q=D=100$, and $u_j=\sigma _{yy}\big ( x \frac{j}{99},\, L\big ),\,j=0,\,\dots ,\,99$. To generate the multi-fidelity approximations for this field, we use the same number of high-fidelity simulations as the previous case, that is $N=30$. The results are shown in Fig. 4. In Fig. 4a we show the low-, high-, and multi-fidelity traction fields for four test cases, where it is clearly seen that the multi-fidelity solution is significantly more accurate that its low-fidelity counterpart. In Fig. 4b we have plotted the histograms of the errors $e^i$, defined in an analogous way for both the SpecMF and low-fidelity data as,

5 $$\begin{aligned} e^i = \frac{||\varvec{w}^i - \varvec{u}^i||_2}{ E(||\varvec{u}^i||_2|)} \times 100\%, \quad i=1,\,\dots ,\,N_{val}, \end{aligned}$$

where the index *i* denotes the *i*-th validation sample, $||\cdot ||_2$ is the $l_2$ norm, and $E(\cdot )$ denotes the mean over all test samples. We observe that the error distribution for the multi-fidelity data is closer to zero, and that there is almost no overlap between the low- and multi-fidelity error distributions. The mean error for the low-fidelity data is around 7% whereas for the multi-fidelity data it is around 1.5%. These values are also reported in Table 2. We note that this example demonstrates that the proposed method can be employed for constructing multi-fidelity approximations of fields, with large values of *D*.

### Aerodynamic coefficients for a family of NACA airfoils

#### Problem description

The multi-fidelity approach is used to tackle a problem in aerodynamics where the goal is to predict the lift, drag and pitching moment coefficients for a family of airfoils operating at different conditions. We consider the 4-digit NACA airfoils, whose shape is defined by three geometric parameters, and investigate how the aerodynamic performance of these airfoils changes at different Reynolds numbers and angles of attack.

#### Parameters and quantities of interest

The parameters of the problem comprise both design and operating condition variables. They are the maximum camber of the airfoil $\eta$, the distance of the maximum camber from the leading edge $x_\eta$, the thickness of the airfoil *t*, the angle of attack $\alpha$, and the Reynolds number $Re = \frac{U c}{\nu }$ (see Fig. 2b). Here, *U* is the flow speed, *c* is the chord of the airfoil, and $\nu$ is the kinematic viscosity of the fluid. In our analysis, the Reynolds number is varied by changing the flow speed *U*. The range of each parameter is reported in Table 1.

The quantities of interest are the aerodynamic coefficients $C_L,\,C_D$, and $C_M$, defined as,

6 $$\begin{aligned} C_L = \frac{L}{\frac{1}{2}\rho U^2 c}, \qquad C_D = \frac{D}{\frac{1}{2}\rho U^2 c}, \qquad C_M = \frac{M}{\frac{1}{2}\rho U^2 c^2}, \end{aligned}$$

where $L,\,D$ and *M* are the lift, drag and the pitching moment about a point located at quarter chord from the leading edge, respectively, and $\rho$ is the density of the fluid. Hence, the vector of quantities of interest is $\varvec{q}(\varvec{\mu }) = [C_L, \, C_D, \, C_M]$, with input parameters $\varvec{\mu }=[\eta ,\, x_\eta ,\, t,\, \alpha ,\, Re]$. The data space is formed by the output quantities of interest only, i.e. $\varvec{u}(\varvec{\mu }) = \varvec{q}(\varvec{\mu })$. A different case, where the Reynolds number is included in the data space, is analyzed in Supplementary Appendix H.

#### Low- and high-fidelity models

The low-fidelity data is generated using XFOIL^37^, a code based on the vortex panel method for the analysis of subsonic airfoils. In this case, the lift and moment coefficients are calculated by direct integration of surface pressure, whereas the drag is recovered by applying the Squire–Young formula^38^. To generate the low-fidelity results, the surface of each airfoil is discretized with 40 panels, and the Reynolds and Mach numbers are set by using a kinematic viscosity of $\nu =10^{-5} \, \mathrm {m^2\,s^{-1}}$ and speed of sound $c_{\textrm{s}} = 340 \; \mathrm {m \; s^{-1}}$.

High-fidelity results are generated via 2D Reynolds-averaged Navier–Stokes (RANS) simulations with a $\textrm{SST} \;\; k-\omega$ turbulence model^39^ using OpenFOAM. The computational domain is a cuboid of dimension $1000 c \times 1000 c \times c$. A hybrid mesh is employed, comprising a C-grid structured mesh in the proximity of the airfoil of size $4c \times 6c$, and an unstructured mesh in the rest of the domain (see Supplementary Appendix G). The number of finite volumes in the mesh varies between 100,000 and 800,000, depending on the Reynolds number. At the outer boundary, Dirichlet boundary conditions for both velocity and pressure are prescribed, while on the airfoil surface a no-slip condition for velocity and zero-gradient condition for pressure are applied. The turbulence intensity of the flow at the outer boundary is set to 2%.

#### Numerical results

**Figure 5:**
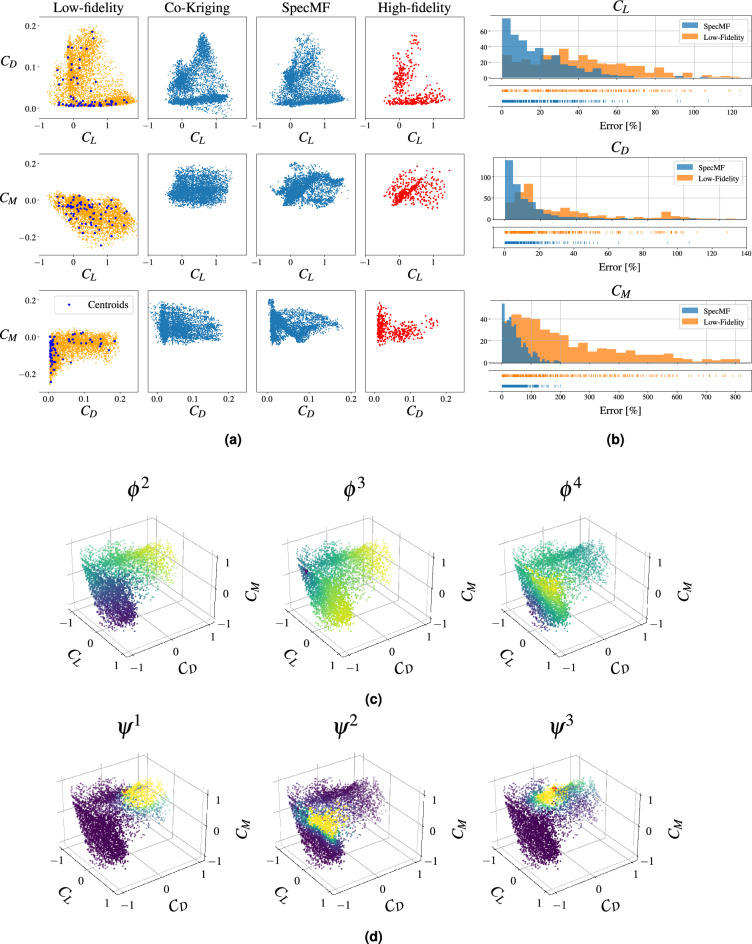
Results for the airfoil problem. (**a**) Left: low-fidelity data with points closest to cluster centroids (in blue). Center: multi-fidelity data points obtained with co-kriging and our multi-fidelity approach (SpecMF). Right: high-fidelity data points used for validation. (**b**) Error distribution for the low- and multi-fidelity data. (**c**) The first three non-trivial eigenfunctions. (**d**) Three typical influence functions.

We sample $\bar{N}=5400$ instances of the input parameter vector from a multivariate uniform distribution and employ XFOIL to generate the set of low-fidelity data (shown in the first column of Fig. 5a in three independent planes). Then, we construct the graph Laplacian and compute its eigendecomposition. The first three non-trivial eigenfunctions are shown in Fig. 5c in the normalized 3-dimensional data space. We embed the low-fidelity data points in the eigenfunction space, and use K-means clustering to find $N=70$ clusters and locate the points closest to their centroids (shown as blue dots in the first column of Fig. 5a). Thereafter, we run CFD simulations to acquire the high-fidelity data at these points. We run $N_{val}=400$ additional high-fidelity simulations, corresponding to randomly selected low-fidelity data points, to be used as a validation set to quantify the performance of the multi-fidelity models. These data points are shown in the fourth column of Fig. 5a.

We solve the minimization problem (17) to determine the transformation (14) which yields the SpecMF data points. The hyper-parameters for this problem are reported in Supplementary Appendix G. Three typical influence functions plotted over the low-fidelity data set are shown in Fig. 5d. The resulting multi-fidelity data points are plotted in the third column of Fig. 5a. The results obtained using co-kriging are plotted in the second column of this figure. When compared to both the low-fidelity and co-kriging data sets, the SpecMF points appear to better represent the structure observed in the high-fidelity data points. This is accomplished by a significant upward shift in the low-fidelity values for the moment coefficient, and a compression in the lift versus drag plane.

The average error for the the low- and multi-fidelity models, as defined in (4), is reported in Table 2. For the SpecMF method, the average error for lift and drag coefficients drops by a factor greater than two, while for the pitching moment it drops more than four times. The co-kriging method reduces the error for the lift and pitching moment only, and not as effectively. The distribution of errors for the low-fidelity and SpecMF data is plotted in Fig. 5d. Once again we observe that the distribution of the error for the SpecMF data is centered closer to zero and displays a narrower spread.

## Discussion

We have proposed a multi-fidelity approach to predict the response of a system when two mechanisms of generating data of different fidelity and cost are available. The method includes three steps: (i) acquire a large number of low-fidelity data, (ii) identify and acquire a small number of key high-fidelity data, and (iii) use the high-fidelity data to improve the accuracy of all low-fidelity data. This is accomplished by constructing an undirected, complete graph from the low-fidelity data and computing its graph Laplacian. The low-lying spectrum of the graph Lalpacian is then used to cluster the low-fidelity data and to determine points closest to the centroids of the clusters. Thereafter, high-fidelity data is acquired only for these select points. This data, along with the spectral decomposition of the graph Laplacian, is used to solve a minimization problem which yields a transformation that maps each low-fidelity data point to new multi-fidelity coordinates. It is shown that this minimzation problem is convex. In numerical experiments, the approach yields multi-fidelity data that is significantly more accurate that its low-fidelity counterpart. In particular, in a problem motivated by biomechanics, this approach improves the accuracy of 1120 low-fidelity data points by a factor of 5–9 (depending on the quantity of interest) by only relying on 30 high-fidelity simulations (less than 3% of the low-fidelity simulations). Similarly, in a problem of aerodynamics, it improves the accuracy of 5400 low-fidelity data points by a factor of 2–4 while only using 70 high-fidelity simulations (1.3% of the low-fidelity simulations). The computational cost of the method scales as $\mathscr{O}(D\bar{N}^2 + N\bar{N})$, where *D* is the dimension of the data space, and $\bar{N}$ and $N$ are the number of low- and high-fidelity data points, respectively. The process of constructing constructing the adjacency matrix involves computing $\mathscr{O}(\bar{N}^2)$ dot products of the differences between *D*-dimensional vectors, and therefore scales as $\mathscr{O}(D\bar{N}^2)$. Further, the cost of computing the low-lying spectrum of the graph Laplacian using iterative methods scales as $\mathscr{O}(\bar{N}^2 + N\bar{N})$^40,41^. However, both these costs can be reduced by setting a cutoff on the number of edges per node on the graph using, for example, an efficient implementation of the $k-$nearest neighbors algorithm^42^. In this case, the cost of the specMF algorithm scales as $\mathscr{O}(k_{\textrm{nn}} D\bar{N}\log \bar{N}+ N\bar{N})$, where $k_{\textrm{nn}}$ is the cutoff on the maximum number of neighbors for every node.

Some limitations and remarks of the proposed method, which also serve to delineate future directions for improvement, are discussed next. In its present form, the choice of the input parameters and output quantities to be included in the data space is somewhat arbitrary. However, it might be possible to develop certain problem-dependent heuristics to identify the parameters and quantities that yield better performance.

The SpecMF method learns the data distribution from the low-fidelity model, and then adjusts it based on a few higher-fidelity data points. Thus, the underlying requirement is that the structure of the low- and high-fidelity data does not change significantly. If the structure arising form the low-fidelity model is fundamentally inaccurate, the benefit from using the method will be limited. We also note that this is a common requirement among most multi-fidelity models. In Supplementary Appendix C, we prove the convergence of the method for the case where the low- and high-fidelity clusters differ by translations. This suggests that the method will perform well when the low- and high-fidelity distributions have the same topology but differ by well-behaved transformations. The graph Laplacian spectrum yields additional insights. A clear spectral gap signifies that the data are effectively clustered, and also provides a way of choosing a suitable number of high-fidelity runs.

Finally, theoretical analysis of the performance of the method in the limit of a large number of high-fidelity data points will lead to a better understanding of its properties.

## Methods

### Background

A complete, weighted graph is a pair $G=(V,\,\varvec{W})$, where $V=\{\varvec{u}^1,\,\dots ,\,\varvec{u}^N\}$ is a set of vertices (or nodes) embedded in $\mathbb {R}^D$, and $\varvec{W}=[W_{ij}]$ is an adjacency matrix. We consider adjacency matrices of the type

7 $$\begin{aligned} W_{ij} = d (|| \varvec{u}^i - \varvec{u}^j ||_2), \end{aligned}$$

where $d(\cdot )$ is a monotonically decreasing function and $||\cdot ||_2$ is the $l_2$ norm. We define the degree matrix $\varvec{D}$ to be a diagonal matrix with

8 $$\begin{aligned} D_{ii} = \sum _{j = 1}^{N} W_{ij}, \end{aligned}$$

and a family of graph Laplacians,

9 $$\begin{aligned} \varvec{L} = \varvec{D}^{-p} (\varvec{D} - \varvec{W}) \varvec{D}^{-q}. \end{aligned}$$

Different choices of *p* and *q* result in different normalizations of the graph Laplacian^43–45^. The graph Laplacian can be used to perform spectral clustering, which amounts to finding an optimal partition of the graph using the spectral properties of $\varvec{L}$^46,47^. The eigenfunctions of $\varvec{L}$ form the set of orthonormal functions from the nodes of the graph to the real numbers $\phi ^{(m)}: V \rightarrow \mathbb {R}$ that solve the eigenvalue problem

10 $$\begin{aligned} \varvec{L}\varvec{\phi }^{(m)} = \lambda _m \varvec{\phi }^{(m)}, \end{aligned}$$

with $\varvec{\phi }^{(m)} = [\phi ^{(m)}_1,\,\dots ,\,\phi ^{(m)}_N]^T$ and $\phi ^{(m)}_i=\phi ^{(m)}(\varvec{u}^i)$. For the un-normalized graph Laplacian ($p=q=0$), the eigenfunctions satisfy the following property,

11 $$\begin{aligned} \varvec{\phi }^{(m)} \cdot \varvec{L} \varvec{\phi }^{(m)}&= \lambda _m \end{aligned}$$

12 $$\begin{aligned}&= \frac{1}{2} \sum _{i,\,j} W_{ij} \left( \phi ^{(m)}_i - \phi ^{(m)}_j \right) ^2. \end{aligned}$$

This implies that eigenfunctions with small eigenvalues provide a mapping of the graph to a line that promotes the clustering of vertices that are strongly connected. Note that the eigenvalue problem (10) admits the trivial solution that maps all vertices to a point, e.g. $\varvec{\phi }^{(1)} = \frac{1}{\sqrt{N}}[1,\,\dots ,\,1]^T$, and has a zero eigenvalue. The eigenfunction corresponding to the smallest non-zero eigenvalue, also called Fiedler vector, represents the non-trivial solution to the problem of embedding the graph onto a line so that connected vertices stay as close as possible^48^. Similarly, the eigenfunctions corresponding to the *k* lowest non-zero eigenvalues, $[\varvec{\phi }^{(2)},\,\dots ,\,\varvec{\phi }^{(k+1)}]$, represent the optimal embedding of the graph into $\mathbb {R}^k$, where the coordinates of a vertex $\varvec{u}^i$ are given by $\varvec{\xi }_i = [\phi _i^{(2)},\dots ,\phi _i^{(k+1)}]$.

### Spectral Multi-Fidelity method (SpecMF)

The three steps to construct the multi-fidelity data are: (1) generate a dense collection of low-fidelity data points, (2) identify key input parameter values at which to acquire the more expensive high-fidelity data, and finally (3) combine the low- and high-fidelity data to construct a multi-fidelity model. Below, we describe each step in detail.

#### Step 1: construction of the low-fidelity graph

We begin by sampling $\bar{N} \gg 1$ points in the parameter space from a simple prescribed probability density to generate the set $\bar{S}=\{\varvec{\mu }^i\}_{i=1}^{\bar{N}}$. Then, we generate the low-fidelity data points $\bar{\varvec{u}}^{i}= \bar{\varvec{u}}(\varvec{\mu }^i), i = 1, \dots , \bar{N}$, and collect them in the set $\bar{\mathcal {D}}=\left\{ \bar{\varvec{u}}^{i}\right\} _{i=1}^{\bar{N}}$. For uniformity, we scale them so that each component lies within $[-1,\, 1]$. We apply the same scaling factors to the high-fidelity data collected in Step 2.

We treat each data point as the vertex of an undirected, complete, weighted graph, and exploit the useful properties of the associated matrices. Hence, we construct a graph with $\bar{\varvec{u}}^{i}$ as vertices, and with weights given by the entries of the adjacency matrix (7) where $d(\cdot )$ is chosen to be a Gaussian kernel,

13 $$\begin{aligned} d(r) \equiv \exp (-r^2/ \sigma ^2). \end{aligned}$$

In the equation above, $\sigma$ is a characteristic scale. It can be treated as a hyperparameter, or its value can also be determined for each vertex by analyzing the statistics of its neighborhood^49^. From the adjacency matrix, we construct the diagonal degree matrix $\varvec{D}$ and a graph Laplacian $\varvec{L}$, using (8) and (9), respectively. For the applications in this paper, we have employed a normalized graph Laplacian with $p,\,q=0.5$ and determined $\sigma$ using the approach described in Ref.^49^.

#### Step 2: selection strategy

Next, we describe the strategy for selecting $N\ll \bar{N}$ nodes for which high-fidelity data is acquired. These nodes are chosen to be close to the centroids of the clusters associated with the low-fidelity data $\bar{\mathcal {D}}$. To find these, we compute the eigendecomposition of the graph Laplacian and leverage the properties of its low-lying spectrum. We embed each data point into the eigenfunctions space and apply a standard clustering algorithm (e.g. K-means) to determine the clusters and their centroids. This is accomplished by,

1. Compute the low-lying eigenfunctions of the graph Laplacian, $\varvec{\phi }^{(m)},\,m=1,\,\dots ,\,K$, with $K = 3 N$. During the selection strategy step (Step 2) we use only the first $N$ of these eigenfunctions. However, in the multi-fidelity transformation step (Step 3) we utilize all of the *K* eigenfunctions.
2. For every low-fidelity data point, $\bar{\varvec{u}}^{i}$, compute the coordinates in the eigenfunction space $\varvec{\xi }^i \in \mathbb {R}^{N}$. These are given by $\varvec{\xi }^i = [\phi _i^{(1)},\dots ,\phi _i^{(N)}], \; i = 1, \dots , \bar{N}$.
3. Use K-means clustering on the points $\{\varvec{\xi }^i\}_{i = 1}^{\bar{N}}$ to find $N$ clusters.
4. For each cluster, determine the centroid and the low-fidelity data point closest to it.
5. Re-index the low-fidelity data set $\bar{\mathcal {D}}$ and the parameters set $\bar{S}$ so that the points identified above correspond to the first $N$ points.
6. Acquire high-fidelity data at the parameter values corresponding to these points, and assemble the data set $\mathcal {D}=\left\{ \varvec{u}^{i}\right\} _{i=1}^{N}$, with $\varvec{u}^{i}= \varvec{u}(\varvec{\mu }^i)$. Note that the elements of $\mathcal {D}$ are the high-fidelity counterparts of the first $N$ elements of $\bar{\mathcal {D}}$.
7. Scale the high-fidelity data with the same scaling factors used in Step 1 for the low-fidelity data.

#### Step 3: multi-fidelity transformation

In this step we generate a multi-fidelity approximation $\left\{ \varvec{w}^{i}\right\} _{i=1}^{\bar{N}}$ that learns the data distribution from the low-fidelity data set and uses the select high-fidelity data to transform this distribution. The proposed multi-fidelity approach seeks a transformation that maps every low-fidelity data point to a new location in the data space, where the displacements are weighted sums of the $N$ known displacements of the select points at which the high-fidelity counterpart is known. That is,

14 $$\begin{aligned} \varvec{w}^{i}= \bar{\varvec{u}}^{i}+ \sum _{j = 1}^{ N} (\varvec{u}^{j}- \bar{\varvec{u}}^{j}) \psi ^{(j)}_i, \qquad i=1,\,\dots ,\,\bar{N}. \end{aligned}$$

Here $\varvec{w}^{i}$ are the multi-fidelity data points, $\varvec{u}^{j}- \bar{\varvec{u}}^{j}$ is the displacement vector that maps the *j*-th low-fidelity point to its high-fidelity location, and $\psi ^{(j)}_i, j = 1, \dots , N$, are the influence functions that determine the effect of the *j*-th displacement vector on the *i*-th point. We require the influence functions to encode the structure of the low-fidelity data distribution, and therefore a natural choice is to write them in terms of the eigenfunctions of the graph Laplacian. For consistency, we also require the influence functions to be a partition of unity. These requirements are satisfied by applying a softmax activation to a set of auxiliary functions $v^{(j)}_i$ that are constructed from a linear combination of the low-lying eigenfunctions of the graph Laplacian. In particular, the influence functions are given by

15 $$\begin{aligned} \psi ^{(j)}_i = \frac{\exp {(v^{(j)}_i)}}{ \sum _{k = 1}^{N} \exp {(v^{(k)}_i})}, \end{aligned}$$

and the auxiliary functions $v^{(j)}_i$ are,

16 $$\begin{aligned} v^{(j)}_i = \sum _{m = 1}^{K} \alpha _{jm} \phi ^{(m)}_i. \end{aligned}$$

The parameter $\alpha _{jm}$ determines the contribution of the *m*-th eigenfunction to the *j*-th auxiliary function, and *K* denotes the cutoff in the spectrum of the graph Laplacian. This cutoff should be proportional to the number of high-fidelity data points. A suggested value, which is used in this study, is $K = 3 N$.

The parameters $\varvec{\alpha }=\{ \alpha _{jm} \}$ are determined by solving the minimization problem

17 $$\begin{aligned} \varvec{\alpha }^* = \arg \min _{\varvec{\alpha }} \, \textsf{J} (\varvec{\alpha }), \qquad \textsf{J} (\varvec{\alpha }) = \textsf{J}_{\textrm{data}}(\varvec{\alpha }) + \omega \textsf{J}_{\textrm{reg}}(\varvec{\alpha }), \end{aligned}$$

with

18 $$\begin{aligned} \textsf{J}_{\textrm{data}}(\varvec{\alpha })&= \frac{1}{N} \sum _{i = 1}^{N} || \varvec{w}^{i}(\varvec{\alpha }) - \varvec{u}^{i}||_2^2, \quad \text{ and } \end{aligned}$$

19 $$\begin{aligned} \textsf{J}_{\textrm{reg}}(\varvec{\alpha })&= \frac{1}{\tau ^2 K N} \sum _{j = 1}^{N} \varvec{v}^{(j)} (\varvec{\alpha }) \cdot ( \varvec{L} + \tau \varvec{I} )^2 \varvec{v}^{(j)} (\varvec{\alpha }). \end{aligned}$$

The first term in (17) is a data misfit term, which forces the multi-fidelity points to be close to the corresponding high-fidelity points. The second term is a structure-preserving term that promotes contributions from eigenfunctions with small eigenvalues. Its form is motivated by a similar term used in semi-supervised learning applications that utilize the graph Laplacian^32^. To examine the effect of this term, we substitute (16) in (19) to find

20 $$\begin{aligned} \textsf{J}_{\textrm{reg}}(\varvec{\alpha }) = \frac{1}{K N} \sum _{j=1}^{N} \sum _{m=1}^{K} \alpha _{jm}^2 \left( 1 + \frac{\lambda _m}{\tau } \right) ^2. \end{aligned}$$

Thus, the values of $\alpha _{jm}$ corresponding to larger values of $\lambda _{m}$ are penalized more. Since the structure of the low-fidelity data is encoded in the eigenfunctions corresponding to the smaller eigenvalues, this term helps in carrying this structure over to the multi-fidelity model. It also makes the proposed algorithm relatively insensitive to the selection of the spectrum cutoff *K*, as the contribution from the higher-order eigenfunctions is weighed less. The presence of $\tau >0$ makes the minimization problem convex and easy to solve^32^, and a good candidate for its value is the smallest non-zero eigenvalue, i.e. $\tau =\lambda _2$. This amounts to solving a problem with a scaled spectrum of the Laplacian.

The regularization constant $\omega$ in (17) balances the interplay between the data misfit term and the structure-preserving regularization term. If its value is too small, the multi-fidelity model is likely to over-fit the high-fidelity data and ignore the structure learned from the low-fidelity data. On the other hand, if its value is too large, the method will yield multi-fidelity points that are significantly different from their high-fidelity counterpart. As described in Supplementary Appendix D, the optimal value of this parameter may be determined using the L-curve method^50^.

Finally, we note that if we do not include the parameters $\varvec{\mu }$ in the definition of $\varvec{u}$, i.e. $\varvec{u}= \varvec{q}(\varvec{\mu })$, they not appear explicitly in any of the equations. This means that the method is insensitive to the dimension of the input space, and it can be applied to a generic point cloud $\{\bar{\varvec{u}}^{i}\}_{i=1}^{\bar{N}}$ embedded in $\mathbb {R}^D$ that has to be transformed based on a few more accurate, or updated, control points $\{\varvec{u}^{i}\}_{i=1}^{N}$, with $N\ll \bar{N}$. This could represent a set of point-wise measurements that are dense in space but not very accurate, for which a smaller number of more precise measurements are available.

### Supplementary Information

Supplementary Information.

## Data Availability

The datasets generated and analyzed in the current study are available from the corresponding author on reasonable request.
