# Supplementary material for: Graph Laplacian-based spectral multi-fidelity modeling
Source: Sci Rep. 2023 Oct 3;13:16618. doi: 10.1038/s41598-023-43719-1 (PMC10547726; doi:10.1038/s41598-023-43719-1)
Supplement: Supplementary file 1 — Supplementary Information. [file 41598_2023_43719_MOESM1_ESM.pdf]

A Gradient of the objective function

In this section, we evaluate an explicit expression for the gradient of the loss function $\mathrm{J} = \mathrm{J}_{\text{data}} + \omega \mathrm{J}_{\text{reg}}$ in Eq. (17) with respect to the parameters $\boldsymbol{\alpha}$.

The gradient of the data misfit term is given by

$$\begin{aligned} \frac{\partial \mathrm{J}_{\text{data}}}{\partial \alpha_{uv}} &= \frac{2}{N} \sum_{i=1}^{N} \left(\boldsymbol{w}^i - \boldsymbol{u}^i \right) \cdot \frac{\partial \boldsymbol{w}^i}{\partial \alpha_{uv}} \\ &= \frac{2}{N} \sum_{i=1}^{N} \left(\boldsymbol{w}^i - \boldsymbol{u}^i \right) \cdot \Big(\sum_{j=1}^{N} \left(\boldsymbol{u}^j - \bar{\boldsymbol{u}}^j \right) \frac{\partial \psi_i^{(j)}}{\partial \alpha_{uv}} \Big) \\ &= \frac{2}{N} \sum_{i=1}^{N} \sum_{j=1}^{N} \left(\boldsymbol{w}^i - \boldsymbol{u}^i \right) \cdot \left(\boldsymbol{u}^j - \bar{\boldsymbol{u}}^j \right) \phi_i^{(v)} \psi_i^{(j)} \left(\delta_{ju} - \psi_i^{(u)} \right). \end{aligned} \tag{A.1}$$

In deriving this expression we have made use of

$$\begin{aligned} \frac{\partial \psi_i^{(j)}}{\partial \alpha_{uv}} &= \frac{\partial}{\partial \alpha_{uv}} \frac{\exp\left(v_i^{(j)}\right)}{\sum_k \exp\left(v_i^{(k)}\right)} \\ &= \frac{\sum_k \exp\left(v_i^{(k)}\right) \cdot \exp\left(v_i^{(j)}\right) \phi_i^{(v)} \delta_{ju} - \exp\left(v_i^{(j)}\right) \exp\left(v_i^{(u)}\right) \phi_i^{(v)}}{\left[\sum_k \exp\left(v_i^{(k)}\right)\right]^2} \\ &= \phi_i^{(v)} \psi_i^{(j)} \left(\delta_{ju} - \psi_i^{(u)} \right), \end{aligned} \tag{A.2}$$

and

$$\frac{\partial v_i^{(j)}}{\partial \alpha_{uv}} = \frac{\partial}{\partial \alpha_{uv}} \sum_{m=1}^{K} \alpha_{jm} \phi_i^{(m)} = \phi_i^{(v)} \delta_{ju}. \tag{A.3}$$

Here δ_{ij} is the Kronecker delta. The gradient of the regularization term is given by

$$\begin{aligned} \frac{\partial \mathrm{J}_{\text{reg}}}{\partial \alpha_{uv}} &= \frac{1}{KN} \sum_{j=1}^{N} \sum_{m=1}^{K} \frac{\partial}{\partial \alpha_{uv}} \alpha_{jm}^2 \left(1 + \frac{\lambda_m}{\tau} \right)^2 \\ &= \frac{2\alpha_{uv}}{KN} \left(1 + \frac{\lambda_v}{\tau} \right)^2. \end{aligned} \tag{A.4}$$

Combining (A.1) and (A.3) we have the expression for the total gradient,

$$\frac{\partial \mathrm{J}}{\partial \alpha_{uv}} = \frac{2}{N} \sum_{i=1}^{N} \sum_{j=1}^{N} \left(\boldsymbol{w}^i - \boldsymbol{u}^i \right) \cdot \left(\boldsymbol{u}^j - \bar{\boldsymbol{u}}^j \right) \phi_i^{(v)} \psi_i^{(j)} \left(\delta_{ju} - \psi_i^{(u)} \right) + \frac{2\omega \alpha_{uv}}{KN} \left(1 + \frac{\lambda_v}{\tau} \right)^2. \tag{A.5}$$

B Positive-definiteness of the Hessian of the objective function

The Hessian can be obtained by differentiating the gradient in (A.1) and (A.3) with respect to the optimization parameters. This yields,

$$\begin{aligned} H_{pquv} &\equiv \frac{\partial^2 \mathrm{J}_{\text{data}}}{\partial \alpha_{pq} \partial \alpha_{uv}} \\ &= \frac{2}{N} \Big[\sum_{i=1}^{N} ||\boldsymbol{w}^i - \boldsymbol{u}^i|| \frac{\partial^2 z^i}{\partial \alpha_{pq} \partial \alpha_{uv}} + \sum_{i=1}^{N} \frac{\partial \boldsymbol{w}^i}{\partial \alpha_{pq}} \cdot \frac{\partial \boldsymbol{w}^i}{\partial \alpha_{uv}} + \frac{\omega}{K} \big(1 + \frac{\lambda_v}{\tau} \big)^2 \delta_{pu} \delta_{qv} \Big], \end{aligned} \tag{B.1}$$

where $z^i \equiv \frac{\boldsymbol{w}^i - \boldsymbol{u}^i}{||\boldsymbol{w}^i - \boldsymbol{u}^i||} \cdot \boldsymbol{w}^i$. In the equation above, the first two terms emerge from the data-misfit term, while the third term is the contribution of the regularization term. The inner product of the Hessian with an arbitrary realization of parameters denoted by $\tilde{\alpha}$ writes

$$\sum_{pquv}\tilde{\alpha}_{pq}H_{pquv}\tilde{\alpha}_{uv}=\frac{2}{N}\Big[\sum_{i=1}^{N}||\boldsymbol{w}^i-\boldsymbol{u}^i||\sum_{pquv}\tilde{\alpha}_{pq}\frac{\partial^2 z^i}{\partial\alpha_{pq}\partial\alpha_{uv}}\tilde{\alpha}_{uv}+\sum_{i=1}^{N}\big(\sum_{pq}\frac{\partial\boldsymbol{w}^i}{\partial\alpha_{pq}}\tilde{\alpha}_{pq}\big)^2+\frac{\omega}{K}\sum_{pq}\big(1+\frac{\lambda_q}{\tau}\big)^2\tilde{\alpha}_{pq}^2\Big]. \tag{B.2}$$

In the equation above, the term $\frac{\partial^2 z^i}{\partial\alpha_{pq}\partial\alpha_{uv}}$ is a symmetric fourth order tensor and therefore has real eigenvalues. We let $\zeta=\lambda_{\min}(\frac{\partial^2 z^i}{\partial\alpha_{pq}\partial\alpha_{uv}}), i=1,\ldots,N$ be the smallest eigenvalue. Therefore, we have

$$\sum_{pquv}\tilde{\alpha}_{pq}H_{pquv}\tilde{\alpha}_{uv}\geq\frac{2}{N}\Big[\zeta\sum_{i=1}^{N}||\boldsymbol{w}^i-\boldsymbol{u}^i||\sum_{pq}\tilde{\alpha}_{pq}^2+\sum_{i=1}^{N}\big(\sum_{pq}\frac{\partial\boldsymbol{w}^i}{\partial\alpha_{pq}}\tilde{\alpha}_{pq}\big)^2+\frac{\omega}{K}\sum_{pq}\tilde{\alpha}_{pq}^2\Big], \tag{B.3}$$

With $\zeta\geq 0$, without any restriction on the data misfit, we have

$$\sum_{pquv}\tilde{\alpha}_{pq}H_{pquv}\tilde{\alpha}_{uv}\geq\frac{2\omega}{NK}\sum_{pq}\tilde{\alpha}_{pq}^2, \tag{B.4}$$

which implies that the Hessain is positive definite. With $\zeta<0$, we require $\sum_{i=1}^{N}||\boldsymbol{w}^i-\boldsymbol{u}^i||\leq\frac{\omega}{2|\zeta|K}$, and use this in (B.3) to arrive at

$$\sum_{pquv}\tilde{\alpha}_{pq}H_{pquv}\tilde{\alpha}_{uv}\geq\frac{\omega}{NK}\sum_{pq}\tilde{\alpha}_{pq}^2, \tag{B.5}$$

Thus, assuming $\sum_{i=1}^{N}||\boldsymbol{w}^i-\boldsymbol{u}^i||\leq\frac{\omega}{2|\zeta|K}$ we conclude that the Hessian is positive definite.

C Solution for a canonical problem

We make the following assumptions on the data:

1. The low-fidelity data $\bar{\boldsymbol{u}}^i$ are distributed among M distinct clusters.
2. $C(i)$ is the cluster number for the $i-$th data point. Without loss of generality, we may assume that points are numbered such that $C(i)=i$ for $i=1,\ldots,M$.
3. For any point i, the high-fidelity data is related to the low-fidelity data via the transformation

   $$\boldsymbol{u}^i=\bar{\boldsymbol{u}}^i+\boldsymbol{\delta}^{C(i)}. \tag{C.1}$$

   where $\boldsymbol{\delta}^j, j=1,\ldots,M$, are the rigid translations for each cluster.
4. For $i=1,\ldots,M$, the high-fidelity data $\boldsymbol{u}^i$ is available and is used to construct the data matching term. Using assumptions 2 and 3, we note that these points,

   $$\boldsymbol{u}^i=\bar{\boldsymbol{u}}^i+\boldsymbol{\delta}^i, \qquad i=1,\ldots,M. \tag{C.2}$$

Under the assumptions above, the proposed method admits a multi-fidelity solution whose error vanishes as the regularization parameter approaches zero.

Proof. In the limit of infinite data, the graph Laplacian of the low-fidelity graph yields M eigenfunctions with null eigenvalues[1] such that,

$$\phi_i^{(j)}=\begin{cases}1, & C(i)=j\\ -1, & C(i)\neq j\end{cases}. \tag{C.3}$$

That is, the j-th eigenfunction attains a value of $+1$ for all points that belong to the j-th cluster and a value of -1 for all other points.

For the proposed method we consider the choice $K=\hat{N}=M$. Further, we consider the special case where the unknown parameters are given by, $\alpha_{jm}=\alpha\delta_{jm}$. With this choice, from Eq. (C.3) and (16) we conclude that

$$v_i^{(j)}=\begin{cases}\alpha, & C(i)=j\\ -\alpha, & C(i)\neq j\end{cases}. \tag{C.4}$$

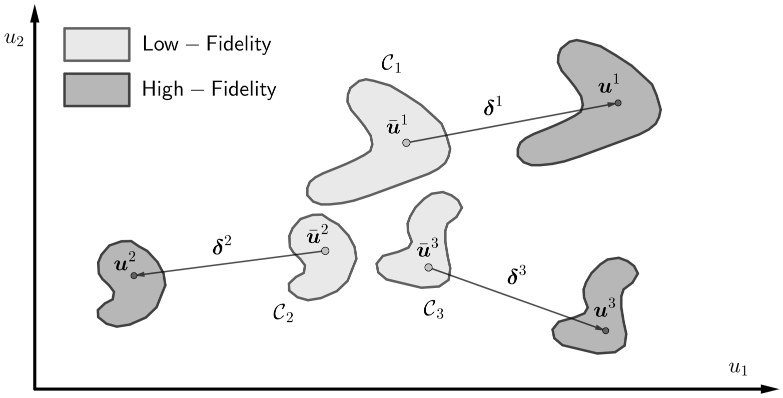

Figure C.1. Schematic of a canonical problem with the data set partitioned into $M = 3$ clusters.

From Eq. (15) this yields,

$$\psi_i^{(j)} = \begin{cases} \frac{1}{1+(M-1)\exp(-2\alpha)}, & C(i) = j \\ \frac{\exp(-2\alpha)}{1+(M-1)\exp(-2\alpha)}, & C(i) \neq j \end{cases}. \tag{C.5}$$

For $i = 1, \ldots, M$, the difference between the multi-fidelity expression (14) and the high-fidelity data (C.2) is given by

$$\begin{aligned} \boldsymbol{w}^i - \boldsymbol{u}^i &= \bar{\boldsymbol{u}}^i + \sum_{j=1}^{M} \boldsymbol{\delta}_j \psi_i^{(j)} - \left(\bar{\boldsymbol{u}}^i + \boldsymbol{\delta}^i\right) \\ &= \boldsymbol{\delta}^i(\psi_i^{(i)} - 1) + \sum_{j=1, j\neq i}^{M} \boldsymbol{\delta}^j \psi_i^{(j)} \\ &= \frac{\exp(-2\alpha)}{1+(M-1)\exp(-2\alpha)} \overbrace{\left(-(M-1)\boldsymbol{\delta}^i + \sum_{j=1, j\neq i}^{M} \boldsymbol{\delta}^j\right)}^{\equiv \boldsymbol{b}^i}, \qquad \text{from (C.5)} \\ &= \gamma(\alpha)\boldsymbol{b}^i. \end{aligned}$$

Where $\gamma(\alpha) \equiv \frac{\exp(-2\alpha)}{1+(M-1)\exp(-2\alpha)}$. Using this expression in the definition of the data matching term (18), we arrive at,

$$\mathsf{J}_{\text{data}}(\alpha) = \gamma(\alpha)^2 \sum_{i=1}^{M} \frac{||\boldsymbol{b}^i||^2}{M}. \tag{C.6}$$

Using (C.4) in Eq. (19) we conclude that the regularization term is given by,

$$\mathsf{J}_{\text{reg}}(\alpha) = \frac{\alpha^2}{M}, \tag{C.7}$$

where we have used the fact the eigenvalues for the eigenfunctions considered in this expansion are zero.

Therefore the total objective function is equal to,

$$\mathsf{J}(\alpha) = \gamma(\alpha)^2 \sum_{i=1}^{M} \frac{||\boldsymbol{b}^i||^2}{M} + \omega \frac{\alpha^2}{M}, \tag{C.8}$$

Further, it is easily verified that

$$\frac{d\gamma}{d\alpha} = 2\gamma\big[(M-1)\gamma - 1\big]. \tag{C.9}$$

Setting $\frac{d\mathsf{J}}{d\alpha} = 0$ to find the stationary point of the objective function, we arrive at,

$$2\gamma\big[(M-1)\gamma - 1\big] \sum_{i=1}^{M} \frac{||\boldsymbol{b}_i||^2}{M} + \omega \frac{\alpha}{M} = 0. \tag{C.10}$$

In the limit $\omega \to 0$, to leading order this equation yields the solution

$$\alpha = -\frac{\ln \omega}{4}. \tag{C.11}$$

Using this solution in the expression of the data matching term we note that $\mathrm{J}_{\mathrm{data}}(\alpha) = O(\omega)$, which tends to zero as $\omega \to 0$. Also $\mathrm{J}_{\mathrm{reg}}(\alpha) = O(\omega^2 \ln^2 \omega)$ which also tends to zero as $\omega \to 0$. Thus both the data matching and regularization terms tend to zero as the regularization parameter tends to zero.

We now show that with choice of α, for any point indexed by i, the coordinates predicted by the multi-fidelity approximation tend to the high-fidelity coordinates as the regularization is reduced. To accomplish this we compute

$$\begin{aligned}
\boldsymbol{w}^i - \boldsymbol{u}^i &= \bar{\boldsymbol{u}}^i + \sum_{j=1}^{M} \boldsymbol{\delta}^j \psi_i^{(j)} - \left(\bar{\boldsymbol{u}}^i + \boldsymbol{\delta}^{C(i)}\right) \\
&= \boldsymbol{\delta}^{C(i)} (\psi_i^{(C(i))} - 1) + \sum_{j=1, j \neq C(i)}^{M} \boldsymbol{\delta}^j \psi_i^{(j)} \\
&= \frac{\exp(-2\alpha)}{1 + (M-1)\exp(-2\alpha)} \left[-(M-1)\boldsymbol{\delta}^{C(i)} + \sum_{j=1, j \neq C(i)}^{M} \boldsymbol{\delta}^j \right], \qquad \text{from (C.5)} \\
&= \gamma(\alpha) \boldsymbol{b}^{C(i)}.
\end{aligned}$$

Therefore,

$$||\boldsymbol{w}^i - \boldsymbol{u}^i|| = \gamma(\alpha) ||\boldsymbol{b}^{C(i)}|| = ||\boldsymbol{b}^{C(i)}|| \omega^{1/2} (1 + O(\omega)). \tag{C.12}$$

Clearly this difference tends to zero as $\omega \to 0$.

D L-curve criterion for determining the regularization parameter

The L-curve is a plot of the data misfit loss $\mathrm{J}_{\mathrm{data}}(\boldsymbol{\alpha}^*(\omega); \omega)$ versus the regularization loss $\mathrm{J}_{\mathrm{reg}}(\boldsymbol{\alpha}^*(\omega); \omega)$ obtained after solving the minimization problem (17) for different values of regularization parameter ω. It can be used to visualize the balance between the two terms and provides a way to tune the regularization parameter. The optimal value ω^* is indeed chosen to be the one corresponding to the elbow of the L-curve, i.e. the point of maximum curvature. In fact, the elbow of the curve separates the regions where the final solution is either dominated by a large data misfit error or by a large regularization loss. Therefore, any perturbation of the parameter ω^* would lead to a significant increase in one loss term or the other. Because the minimization problem (17) is inexpensive to solve, we can use the L-curve criterion to determine the optimal value of ω. In Figure D.1 we show the L-curve used to determine the regularization parameter for the traction on soft material problem in Section 2.1.

E Effect of the selection strategy of high-fidelity data

In Step 2 of the method, we propose a strategy to select the parameters values at which to acquire high-fidelity data. The strategy consists in determining the low-fidelity data points that are the closest to the centroids of the clusters arising within the low-fidelity data, and then employ the high-fidelity model to compute their counterpart.

We want to compare the performance of this strategy against a random selection of the low-fidelity points for which we acquire a high-fidelity version. To do that, we consider the problem of predicting the traction on a soft body and the results obtained in Section 2.1. Then, we solve the same problem with a random selection of the parameters at which to compute high-fidelity data points. The number of high-fidelity data points used is fixed, $N = 30$, and the problem is solved 200 times with different random selections. Then, we compute the mean and standard deviation of the error for each component as defined in (4), and display them in Table E.1.

We notice that the errors committed with a random selection is larger than the errors attained with the proposed selection approach, but is still considerably smaller than the low-fidelity errors. This suggests that the proposed selection strategy ought to be preferred to a random choice when possible, but that the multi-fidelity method can be successfully applied even when the set of high-fidelity data is predetermined.

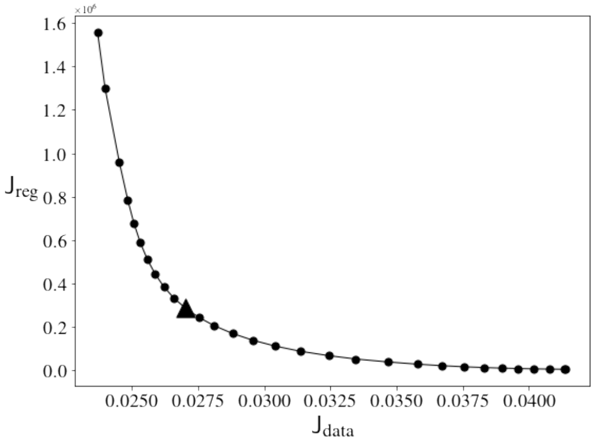

Figure D.1. L-curve for the traction problem in Section 2.1. The optimal value for the regularization parameter is the one corresponding to the elbow of the curve, marked with a triangle in the graph.

ERROR [%]	SOFT BODY WITH INCLUSION				
Quantity of interest	t_1	t_2	t_3	t_4	σ_{yy}^{max}
Low-fidelity	4.48	7.15	7.21	4.65	10.19
SpecMF + proposed selection strategy	**0.52**	**1.2**	**1.06**	**0.51**	**2.0**
SpecMF + random selection	0.63 (0.11)	1.54 (0.16)	1.42 (0.16)	0.81 (0.12)	2.38 (0.18)

Table E.1. Errors in the multi-fidelity model constructed with the proposed and random selection strategies. For the random strategy, we report the mean and the standard deviation (in parenthesis).

F Traction on a soft material with a stiff inclusion

To generate the multi-fidelity data set, we selected a value of $\tau = 3 \cdot 10^{-10}$, which corresponds to the smallest non-zero eigenvalue, and a spectrum cutoff of $K = 90$. To determine the value of the regularization parameter in Eq. (17) we make use of the L-curve method to study the data misfit loss versus the regularization loss for different values of $\omega \in [10^{-9}, 10^{-6}]$ (see Figure D.1). The optimal value corresponding to the elbow of the curve is found to be $\omega^* = 8.53 \cdot 10^{-9}$.

The low- and high-fidelity models used to solve the elasticity problem described in Section 2.1 are finite element solvers that differ by their mesh density, i.e. the number of elements used to discretize the computational domain. In Figure F.1 we show a comparison between the two meshes.

The low- and high-fidelity solutions of the problem show similar trends, but the low-fidelity solutions tend to underestimate the magnitude of traction field. In Figure F.2 we show a sample of low- and high-fidelity solutions for different instances of input parameters.

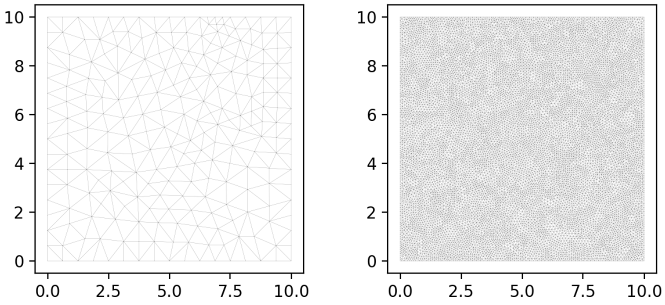

Figure F.1. Comparison between the two meshes of the low- and high-fidelity finite element models used to solve the traction on soft material problem.

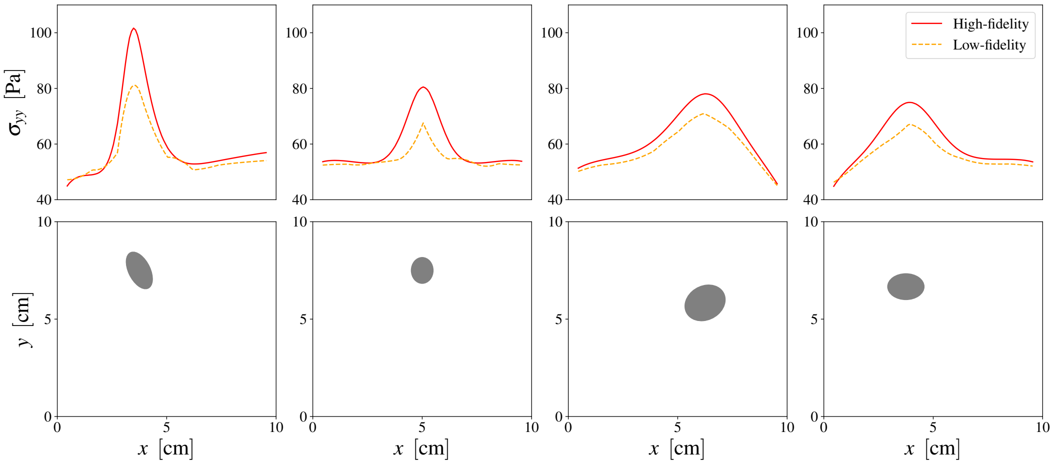

Figure F.2. Sample of low- and high-fidelity traction fields for different inclusions.

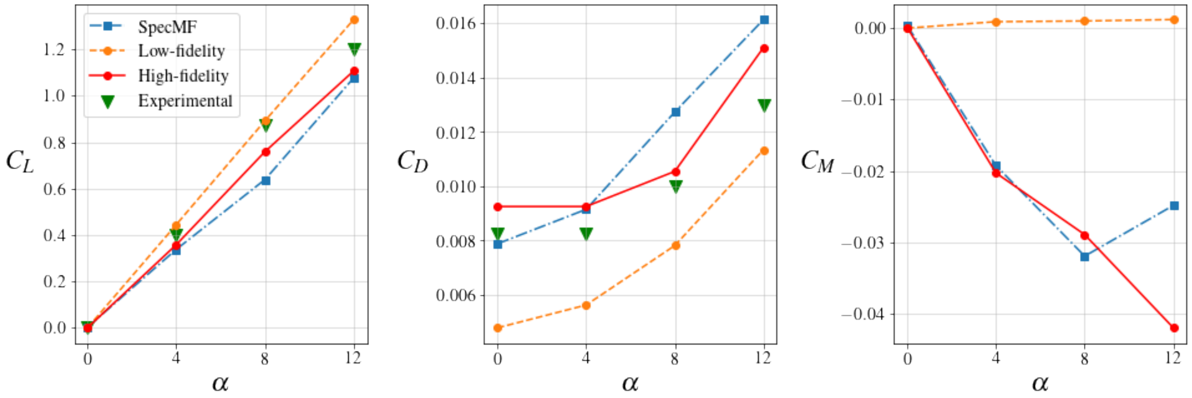

Figure G.1. Comparison of the curves of the lift, drag and moment coefficients versus angle of attack for the airfoil NACA 0012 at $Re = 6 \cdot 10^6$. The low-fidelity data are indicated with a dashed orange line, the multi-fidelity data with a dash-dot blue line, the high-fidelity data with a solid red line, and the experimental data are marked with a green upside-down triangle.

G Aerodynamic coefficients for a family of NACA airfoils

In Section 2.2 we consider the problem of predicting the aerodynamic coefficients of 4-digit NACA airfoils for different Reynolds number and angles of attack. The multi-fidelity transformation is found with a spectrum cutoff of $K = 210$, a value of $\tau = 9.07 \cdot 10^{-4}$, and a regularization parameter $\omega^* = 8.87 \cdot 10^{-7}$. The resulting multi-fidelity data points are shown in Figure 5a as a point cloud in the 3-dimensional data space of the lift, drag and moment coefficients (C_L, C_D and C_M, respectively).

To better visualize the data at the three levels of fidelity, we select the case of the NACA 0012 airfoil profile at Reynolds $Re = 6 \cdot 10^6$. In Figure G.1 we plot the graphs of the aerodynamic coefficients versus angle of attack for the three models, including experimental data available for lift and drag coefficients[2]. We observe that the high-fidelity CFD simulations match the experimental results within 10% error for lift and 15% error for drag. In Figure G.2 we show the computational mesh used to perform these simulations.

By analyzing the lift and drag coefficients curves, we note that the multi-fidelity model could learn and retain the low-fidelity trend, and adjust the magnitudes in light of the sparse high-fidelity data. For the moment coefficient, the low-fidelity data shows a significant disagreement with the high-fidelity results. Nonetheless, the predictions of the multi-fidelity model more closely match the high-fidelity data in magnitude. The prediction of the trend is also correct for angles of attack smaller than 8 degrees. It is verified that none of the high-fidelity data points related to the NACA 0012 airfoil were used in constructing the multi-fidelity model. We conclude that the multi-fidelity model could correct the data structures in the 3-dimensional data space using the few select high-fidelity data points available, and has increased the accuracy for all low-fidelity points.

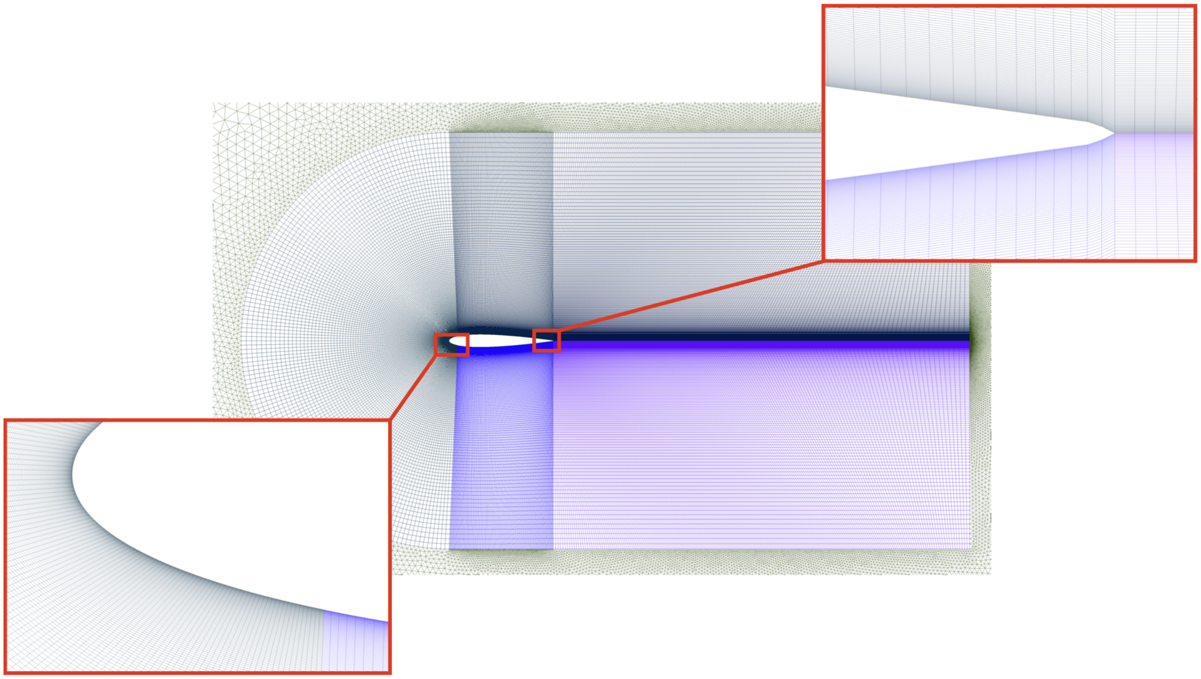

Figure G.2. Example of a mesh used for the high-fidelity CFD simulations.

H Definition of the data space

As discussed in Section 4.2, we consider problems where the solution for a given instance of input parameters $\boldsymbol{\mu}$ is a set of quantities of interest $\boldsymbol{q}(\boldsymbol{\mu})$.

The data space $\boldsymbol{u}$ where the low-fidelity graph is constructed is defined as $\boldsymbol{u}(\boldsymbol{\mu}) = \boldsymbol{R}(\boldsymbol{\mu}, \boldsymbol{q}(\boldsymbol{\mu}))$, with $\boldsymbol{R}$ being a restriction operator that extracts the appropriate components of $\boldsymbol{\mu}$ and $\boldsymbol{q}$. The choice of $\boldsymbol{R}$ is problem dependent. It should include all the variables directly related to the predictions one wants to make, i.e. the subset of the relevant components of $\boldsymbol{q}$, and the parameters that would help identifying clusters and structures in the data.

For example, including bifurcation parameters in the data space is important, as small changes in their value can cause significant changes in the topology or qualitative nature of the physical solution. Similarly, parameters that identify different and distinct regimes of the solution, e.g. the Reynolds number in fluid dynamics, provide valuable information about different data points, and can help separating the point cloud. This is especially important when the performance of the low-fidelity model strongly depend upon certain parameters. If the error of the low-fidelity data significantly differs based on a parameter, adding it to the data space will make sure that the transformation (14) will act accordingly.

In Figure H.1 we show a schematic of a simple case where adding the parameter μ_1 to the data space (q_1, q_2) leads to a clear separation of the data points. In this illustrative example, including the parameter in the data space allows for a better selection of the high-fidelity data to acquire and a more appropriate treatment of the two clusters.

It is important to notice that the transformation (14) does not lead to any displacements along the parameters directions, as the high-fidelity data points will have by construction the same input parameters. Hence, the multi-fidelity transformation will act only on the space of the quantities of interests, as it should.

H.1 Numerical results with augmented data space

In this Section we analyze the results of the numerical problems proposed in Section 2, when the input parameters are included in data space.

For the problem of traction on soft body (Section 2.1), with input parameters $\boldsymbol{\mu} = [x_c, y_c, \theta, a, b]$ and quantities of interest $\boldsymbol{q} = [f_1, f_2, f_3, f_4, \sigma_{yy}^{\max}]$, we consider the case where the data space is formed by concatenating all inputs and all outputs, i.e. $\boldsymbol{u} = [\boldsymbol{\mu}, \boldsymbol{q}]$.

On the other hand, for the problem of the aerodynamic coefficients of NACA airfoils (Section 2.2), the input parameters and the set of quantities of interest are $\boldsymbol{\mu} = [\eta, x_\eta, t, \alpha, Re]$ and $\boldsymbol{q} = [C_L, C_D, C_M]$, respectively. In this case, we include only the Reynolds number in the data space, i.e. $\boldsymbol{u} = [Re, C_L, C_D, C_M]$.

For both problems, we apply once again the proposed multi-fidelity method considering the graph constructed in the

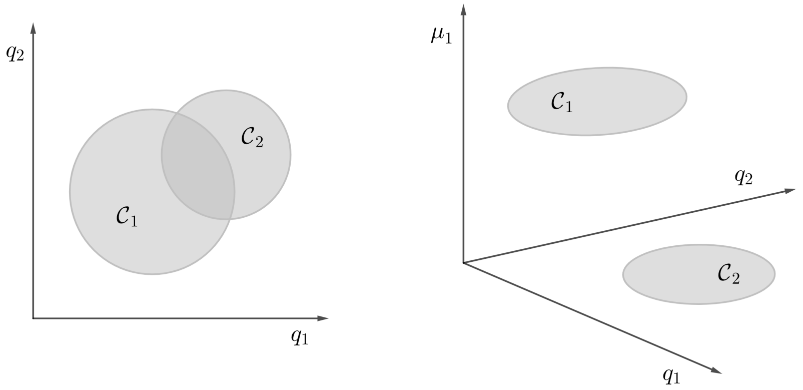

Figure H.1. Schematic of a case where adding the parameter μ_1 to the data space (q_1, q_2) separates the data set into two distinct clusters, $\mathscr{C}_1$ and $\mathscr{C}_2$.

augmented data space. The numerical results are reported in Table H.1. We note that in these particular numerical experiments, using only the quantities of interest in the definition of the data space (that is $\boldsymbol{u} = \boldsymbol{q}$) leads to better results in almost all cases.

ERROR [%]	SOFT BODY WITH INCLUSION					AIRFOIL		
Quantity of interest	f_1	f_2	f_3	f_4	$\sigma_{yy}^{\max}$	C_L	C_D	C_M
Low-fidelity	4.48	7.15	7.21	4.65	10.19	40.40	28.84	216.96
SpecMF ($\boldsymbol{u} = \boldsymbol{q}$)	**0.52**	**1.24**	**1.07**	**0.51**	2.02	**18.56**	**10.77**	**45.95**
SpecMF (augmented data space)	0.65	1.52	1.56	0.63	**1.87**	20.01	12.04	49.62

Table H.1. Error of the low- and multi-fidelity model for each quantity of interest.

References

1. Hoffmann, F., Hosseini, B., Ren, Z. & Stuart, A. M. Consistency of semi-supervised learning algorithms on graphs: Probit and one-hot methods. *J. Mach. Learn. Res.* **21**, 186:1–186:55 (2020).

2. Ladson, C. L. Effects of independent variation of mach and reynolds numbers on the low-speed aerodynamic characteristics of the naca 0012 airfoil section. *Washington, D.C: Natl. Aeronaut. Space Adm. Sci. Tech. Inf. Div.* (1988).
